# Editorial: Cognitive Brain-Inspired Cyber-Physical Systems in Industrial Informatics

**DOI:** 10.3389/fnbot.2022.926538

**Published:** 2022-06-23

**Authors:** Arun Kumar Sangaiah, Patrick Siarry

**Affiliations:** ^1^School of Computer Science and Engineering, VIT University, Vellore, India; ^2^Department of Industrial Engineering and Management, National Yunlin University of Science and Technology, Douliu, Taiwan; ^3^Laboratoire Images, Signaux et Systèmes Intelligents, Université Paris-Est Créteil Val de Marne, Créteil, France

**Keywords:** cognitive system, cyber physical system (CPS), industrial informatics, brain-inspired computation, cognitive cyber-physical systems

## Introduction

Artificial intelligence (AI), cognitive computing, machine learning, and brain-inspired computing are among the most trending research focuses in cyber physical systems (CPSs). Brain inspiration computing leads to faster more efficient threat detection in CPSs. This has resulted in the development of brain-inspired computing algorithms including nature-inspired algorithms, genetic algorithms, swarm algorithms, and pattern recognition algorithms, for addressing hard computational problems and CPS research challenges. Cognitive cyber-physical systems (CCPSs) are witnessing a rapid transformation as an interdisciplinary technology that blends physical components and computing devices to enable AI-based solutions. CCPS is integrating machine learning/AI with brain-inspired computing in realizing smart systems. This era is witnessing a rapid transformation in digital technology, AI with brain-inspired computing-based solutions will play a vital role in industrial informatics. The application of CCPSs with brain-inspired computing in the industrial revolution of Industry 4.0 has made smart factories in the industrial evolution. Other applications like smart household devices, medical systems, autonomous driving systems, robotic systems use in connected devices, data analytics, cloud computing, and artificial intelligence automate the process further. These enabling technologies have full capabilities to provide interoperability, information transparency, technical assistance, and decentralized decisions. The fifth industrial revolution is expected to pair humans and machines to further utilize human brainpower and creativity to increase process efficiency by combining workflows with intelligent systems. In Industry 5.0, robots will not only be a programmable machine but will also transform into an ideal human companion for some scenarios. With recent enlargements in brain inspiration computing and AI, it is now possible to create even more realistic and brain intelligence level algorithms. The purpose of this editorial is to explore the research output in enabling CPS technologies and their state-of-the-art approaches, introduce recent advances, and, therefore, show the potential of brain inspiration computing in CCPSs. In this special issue, the published papers have shown comprehensive overviews/methodological approaches to brain-inspired learning and computing, with a focus on applications of interest to CCPSs.

## Papers in This Special Section

The first paper authored by Senthilkumar et al. proposed a methodology for a remote authentication scheme with smart card–based healthcare information using the ECC algorithm. The authors have performed an informal analysis to substantiate the claim that the proposed scheme provides sufficient security while maintaining usability. Maintaining user anonymity also ensures that others cannot access data without prior approval of the file owner. It also supports file integrity with the help of an elliptic curve digital signature algorithm. Furthermore, this research shows that the proposed protocol is effective in terms of the communication and computation cost in secure cloud storage of smart card–based healthcare information.

In the next paper by Huang et al., they employed a CNN algorithm to realize the early prediction of refractory epilepsy in children. The results showed that after data preprocessing, CNN can predict and diagnose early refractory epilepsy in children accurately, and had a favorable effect on magnetic resonance imaging (MRI) processing of the patient's brain. This algorithm has high guiding significance in the early diagnosis and treatment of refractory epilepsy in children, and it is worthy of clinical adoption. However, this work does not subdivide the types of refractory epilepsy in children and does not evaluate the specific effects of the proposed algorithm to assist in the treatment.

The third paper by Li et al. has established a CNN algorithm model and applied it to the MRI image segmentation of patients with acute cerebral infarction to evaluate the effect of butylphthalide combined with edaravone treatment on the neurological function of patients. The results showed that MRI images based on the CNN algorithm could clearly show the lesions of patients with acute cerebral infarction. In addition, it was observed that after the treatment of butylphthalide combined with edaravone, the lesion of cerebral infarction became smaller and the condition of artery stenosis gradually improved. These results indicated that CNN-based MRI image segmentation had a high application value in the evaluation of acute cerebral infarction after drug treatment. However, the shortcomings of this study are that the included sample size is small, the source is a single center, and the classification and comparison of patients with acute cerebral infarction at different stages are not completed.

In the last paper by Peng et al., they proposed a novel OCT-based CCA method for target identification to perform reaching tasks of a 7-DOF robotic arm in the 3D space. An offline experiment and online experiment were designed to confirm the improvements of the OCT-CCA method and the control performance of the robotic arm in the 3D space. The offline comparison results demonstrated that the classification accuracy of the OCT-CCA method outperforms the CCA and IT-CCA methods regardless of the time window lengths. The online experiment was completed by a controlled 7-DOF robotic arm. By focusing gaze on the flickering targets corresponding to the control command, subjects can manipulate the robotic arm as desired. The results showed that all five subjects can complete the designated reaching tasks within the appropriate time window. The success of the online experiment with five subjects demonstrates the simplicity and flexibility of the robotic arm control system in SSVEP-based BCI, which will be a practicable and promising application for disabled individuals in their daily lives.

## Author Contributions

Both authors listed have made a substantial, direct, and intellectual contribution to the work and approved it for publication.

## Conflict of Interest

The authors declare that the research was conducted in the absence of any commercial or financial relationships that could be construed as a potential conflict of interest.

## Publisher's Note

All claims expressed in this article are solely those of the authors and do not necessarily represent those of their affiliated organizations, or those of the publisher, the editors and the reviewers. Any product that may be evaluated in this article, or claim that may be made by its manufacturer, is not guaranteed or endorsed by the publisher.

